# Dynamic Changes in Mimic Muscle Tone During Early Orthodontic Treatment: An sEMG Study

**DOI:** 10.3390/jcm14145048

**Published:** 2025-07-16

**Authors:** Oskar Komisarek, Roksana Malak, Paweł Burduk

**Affiliations:** 1 Department of Otolaryngology, Phoniatrics and Audiology, Faculty of Medicine, Collegium Medicum, Nicolaus Copernicus University in Toruń, 87-100 Toruń, Poland; pburduk@cm.umk.pl; 2 Department and Clinic of Rheumatology, Rehabilitation and Internal Diseases, Poznan University of Medical Sciences, 61-545 Poznań, Poland; rmalak@ump.edu.pl

**Keywords:** surface electromyography, facial muscles, orthodontic treatment, fixed appliances, neuromuscular adaptation, facial esthetics

## Abstract

**Background:** Surface electromyography (sEMG) enables the non-invasive assessment of muscle activity and is widely used in orthodontics for evaluating masticatory muscles. However, little is known about the dynamic changes in facial expression muscles during orthodontic treatment. This study aimed to investigate alterations in facial muscle tone during the leveling and alignment phase in adult female patients undergoing fixed appliance therapy. **Methods:** The study included 30 female patients aged 20–31 years who underwent sEMG assessment at four time points: before treatment initiation (T0), at the start of appliance placement (T1), three months into treatment (T2), and six months into treatment (T3). Muscle activity was recorded during four standardized facial expressions: eye closure, nasal strain, broad smile, and lip protrusion. Electrodes were placed on the orbicularis oris, orbicularis oculi, zygomaticus major, and levator labii superioris alaeque nasi muscles. A total of 1440 measurements were analyzed using Friedman and Conover-Inman tests (α = 0.05). **Results:** Significant changes in muscle tone were observed during treatment. During lip protrusion, the orbicularis oris and zygomaticus major showed significant increases in peak and minimum activity (*p* < 0.01). Eye closure was associated with altered orbicularis oris activation bilaterally at T3 (*p* < 0.01). Nasal strain induced significant changes in zygomaticus and levator labii muscle tone, particularly on the right side (*p* < 0.05). No significant changes were noted during broad smiling. **Conclusions:** Orthodontic leveling and alignment influence the activity of selected facial expression muscles, demonstrating a dynamic neuromuscular adaptation during treatment. These findings highlight the importance of considering soft tissue responses in orthodontic biomechanics and suggest potential implications for facial esthetics and muscle function monitoring.

## 1. Introduction

Surface electromyography (sEMG) has evolved dramatically since the pioneering single-channel recordings of the early 20th century. Digital multichannel amplifiers introduced in the 1990s made it possible to quantify jaw-closing muscle activity in vivo, and over the past five years miniaturized wireless and high-density electrode arrays have taken the technique from the laboratory to the dental chair-side [1,2,3].

A targeted PubMed query (May 2025) retrieved >320 articles that applied sEMG to the stomatognathic system between 2020 and 2024, reflecting an annual growth rate of about 14%. Recent systematic reviews confirm its utility in monitoring neuromuscular responses to orthodontic appliances in children and adolescents [4]. Parallel methodological advances such as high-resolution facial mapping [2,3] have provided detailed atlases of voluntary muscle activation that can serve as normative baselines for clinical studies.

Despite this momentum, the resting tone of mimic muscles during the biologically turbulent early phase of fixed appliance therapy remains largely uncharted. These muscles act as functional antagonists to orthodontic forces; elevated baseline activity may compromise arch-wire biomechanics, contribute to unwanted incisor torque, and influence smile esthetics. Contemporary orthodontic trials continue to focus on bracket adhesion [5] or hard-tissue outcomes such as pain and root resorption after surgical acceleration [6], while prospective studies that combine sEMG with three-dimensional risk mapping of miniscrew sites (e.g., nasopalatine canal morphology [7]) are still scarce.

Real-time insight into mimic muscle adaptation could enable personalized adjustments to archwire stiffness and adjunctive physiotherapy, potentially improving both treatment efficiency and patient-reported esthetic outcomes. Integrating sEMG with digital workflow data would also align with current trends toward evidence-based, minimally invasive orthodontics.

Aim and hypothesis. The objective of the present prospective study was to quantify the dynamic changes in the resting root mean square (RMS) activity of selected facial muscles during the first 12 weeks of multibracket treatment in young adults. We hypothesized that with the introduction of light, continuous orthodontic forces would produce a ≥15% reduction in RMS amplitude relative to baseline, reflecting neuromuscular relaxation secondary to occlusal disengagement.

## 2. Materials and Methods

### 2.1. Ethical Approval

This study was conducted in accordance with the Declaration of Helsinki and was approved by the Bioethics Committee of the Poznań University of Medical Sciences (Approval No. 210/19). Written informed consent was obtained from all participants prior to inclusion in the study.

### 2.2. Study Population

Thirty female patients aged 20 to 31 years (mean age: 24.6 years) were recruited from the Department of Maxillofacial Orthopaedics and Orthodontics at Poznań University of Medical Sciences. All participants required fixed appliance orthodontic treatment due to malocclusion. Inclusion criteria were as follows: (1) female sex; (2) age between 20 and 31 years; (3) indication for full-arch fixed appliance treatment; (4) Class I, II, or III skeletal pattern diagnosed with cephalometric analysis; (5) absence of neuromuscular disorders or facial asymmetry; and (6) good general health. Exclusion criteria included the following: (1) congenital craniofacial anomalies, (2) prior orthodontic treatment with fixed appliances, (3) history of craniofacial trauma, (4) presence of orofacial pain or temporomandibular joint dysfunction, (5) habitual mouth breathing.

All participants were informed that participation was voluntary, non-remunerated, and for scientific purposes only.

### 2.3. Sample Size and Power Analysis

A priori sample-size estimation was performed with G*Power 3.1.9.7 (Heinrich-Heine-Universität Düsseldorf, Germany), in accordance with the tutorial by Kang (2021) [8]. Because the present study comprised four repeated measurements in a single cohort, the repeated-measures ANOVA within-subjects option was selected.

Effect size. Pilot recordings obtained from five volunteers during bracket bonding (unpublished) showed a mean 15% decrease (SD ≈ 25%) in the resting RMS activity of the orbicularis oris after 12 weeks. This corresponds to Cohen’s f = 0.25, classified as a medium effect. The f metric was chosen instead of d because it is recommended for power analysis in multi-time point within-subject ANOVA models and incorporates the correlation structure of repeated observations [9].

Parameters. A two-tailed α of 0.05 and statistical power of 0.80 were adopted to balance type-I and type-II error risk in the exploratory clinical research. Assuming a correlation among repeated measures of r = 0.50 and a non-sphericity correction (ε) of 1.0, the calculation indicated a minimum sample of n = 28. To compensate for an anticipated 5% attrition, the target size was rounded up to 30 participants.

TMJ-specific perspective. Guidelines for temporomandibular joint and masticatory muscle research [10] indicate that a cohort of 30 subjects provides more than 90% power to detect large effects (f ≥ 0.40). The final sample therefore satisfies both the medium-effect target derived from pilot data and the large-effect threshold recommended for TMJ-related sEMG studies.

The selected standard deviation (SD ≈ 25%) and effect size (Cohen’s f = 0.25) were based on pilot recordings obtained from five volunteers undergoing bracket bonding, where a 15% change in orbicularis oris RMS was noted. This value reflects expected physiological variability in resting tone and aligns with medium effects typically observed in orthodontic muscle adaptation studies.

### 2.4. sEMG Acquisition and Processing

Bilateral surface EMG signals were recorded with a 16-bit wireless amplifier (TeleMyo DTS, Noraxon USA Inc., Scottsdale, AZ, USA). The sampling rate was 2000 Hz, with an online band-pass filter of 20–450 Hz (fourth-order Butterworth) and a 50 Hz notch filter to suppress mains interference. Electrode–skin impedance was kept below 5 kΩ (maximum accepted 10 kΩ), verified with the device’s built-in impedance tester before each trial.

All recordings were performed between 08:00 and 11:00 a.m. to minimize diurnal variation in facial muscle tone. Each functional task described in lines 105–109 (resting posture, gentle smile, broad smile, lip protrusion) was executed three times with a 30 s rest interval. The entire four-task sequence was then repeated once after a 5 min break, yielding two sets of three trials per task.

Raw sEMG data were analyzed using Noraxon software Version 3.12.70 (Noraxon USA Inc., Scottsdale, AZ, USA). Offline processing replicated the online filter settings (20–450 Hz, zero-lag Butterworth), followed by full-wave rectification and calculation of the root mean square (RMS) envelope using a 250 ms moving window. RMS values were averaged across the three trials of each set and expressed in microvolts.

Reliability analysis employed the two-way mixed-effects, single-measurement, absolute-agreement intraclass correlation coefficient ICC(3,1) [10]. ICC values for resting RMS ranged from 0.82 to 0.93 across muscles, indicating good-to-excellent intra-session repeatability.

### 2.5. Electrode Placement

Electrodes were bilaterally positioned over four facial expression muscles, following the anatomical mapping described by Boxtel [11]:

- orbicularis oris,
- zygomaticus major,
- orbicularis oculi,
- levator labii superioris alaeque nasi.

**Figure 1:**
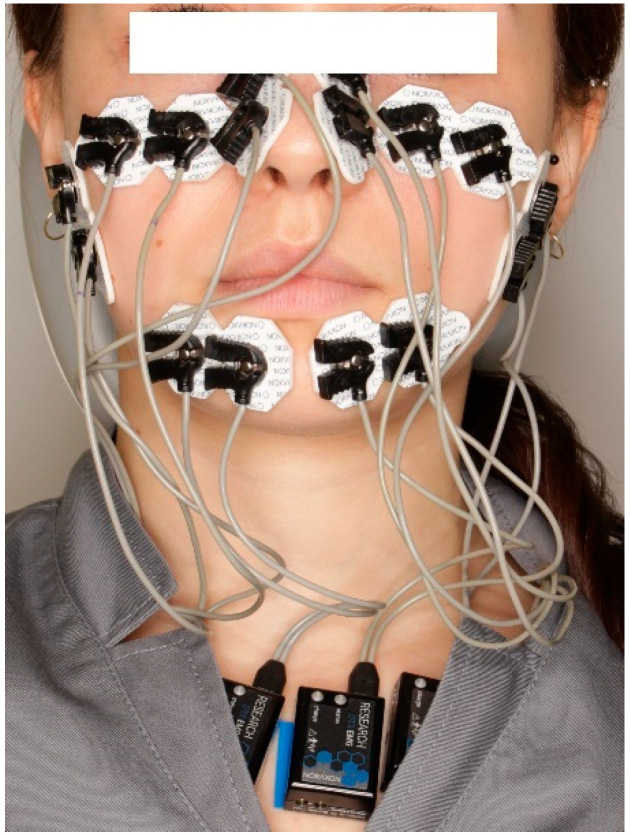
Electrode placement scheme used for surface electromyographic recordings. Electrodes were positioned bilaterally on the orbicularis oris, zygomaticus major, orbicularis oculi, and levator labii superioris alaeque nasi muscles. Placement followed anatomical references described by Boxtel [11].

Placement is illustrated in Figure 1. Electrodes were affixed with consistent inter-electrode spacing and orientation across all subjects.

### 2.6. Experimental Protocol

**Figure 2:**
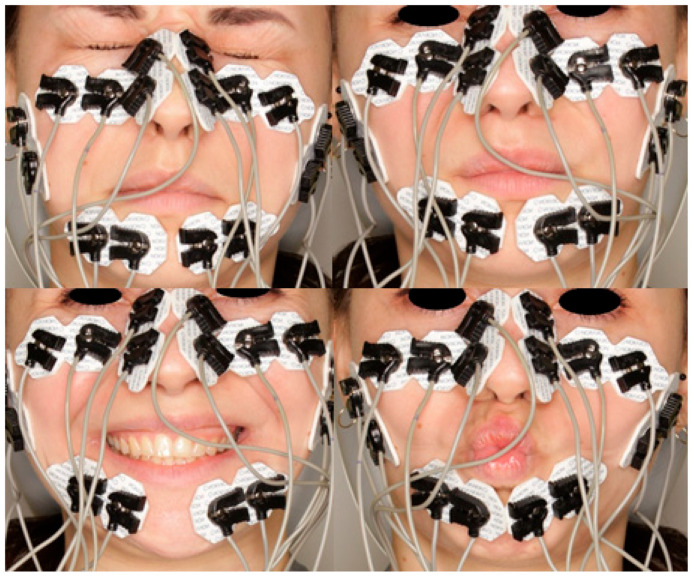
Standardized facial expressions assessed during sEMG recording: eye closure, nasal strain, broad smile, forward lip protrusion. Each expression was performed three times with maximal voluntary effort.

sEMG measurements were performed in a quiet, isolated room to minimize environmental artifacts. Each subject was seated upright, with feet flat on the ground and knees bent at a 90° angle. During each session, the subject performed four standardized facial movements (Figure 2):

1. Eye closure,
2. Nasal strain (wrinkling the nose),
3. Broad smile,
4. Forward lip protrusion.

Movements were performed three times each, with maximal voluntary effort and 30 s rest intervals to prevent muscle fatigue. Each testing session yielded twelve total recordings per subject.

### 2.7. Measurement Time Points

Recordings were obtained at four standardized time points during orthodontic treatment:

- T0—prior to appliance bonding,
- T1—on the day of appliance placement (start of leveling and alignment phase),
- T2—3 months after T1,
- T3—6 months after T1.

### 2.8. Data Processing

The raw root mean square (RMS) signal from each muscle was used for analysis. Amplitude normalization was not performed due to the heterogeneous architecture and recruitment patterns of facial muscles. Unlike limb or masticatory muscles, mimic muscles have variable fiber orientation, superficial positioning, and often lack defined antagonists, which complicates the establishment of a reliable and consistent maximum voluntary contraction (MVC) baseline. Furthermore, standard MVC procedures may introduce bias when evaluating subtle activation changes in small facial muscles with overlapping functions. We therefore chose to analyze raw RMS values to preserve natural intra-muscular variability and dynamic response. However, we acknowledge that this approach may limit intra- and inter-subject comparability and should be interpreted with caution, particularly when comparing between muscle groups.

### 2.9. Statistical Analysis

All statistical analyses were performed using Statistica 14.0 (TIBCO Software Inc., Palo Alto, CA, USA) and PQStat version 1.8.6 (PQStat Software, Poznań, Poland). The significance level was set at α = 0.05. The Shapiro–Wilk test was used to assess the normality of data distribution. For variables following normal distribution, repeated-measures ANOVA was applied to evaluate changes over time. When the distribution deviated from normality, the Friedman test was used, followed by Conover–Inman post hoc comparisons. Differences between the left and right sides of the same muscle were assessed using the paired Student’s t-test for normally distributed variables or the Wilcoxon signed-rank test otherwise.

When appropriate, correlation analysis was performed using Pearson’s r or Spearman’s rank correlation coefficient (Rs), depending on distribution normality. Statistical significance was accepted at *p* < 0.05 or *p*_adj_ < 0.05 after Bonferroni correction for multiple comparisons. Effect sizes were expressed as partial eta-squared (ηp^2^) or Kendall’s W and Cohen’s dz where applicable.

## 3. Results

Thirty female patients completed the study protocol and all scheduled sEMG assessments. The mean age of the cohort was 24.6 years (range: 20–31), with a median of 24.0 years, a lower quartile of 23.0 years, and an upper quartile of 27.0 years. Skeletal pattern analysis revealed that 66.7% of the patients (n = 20) presented with a Class I skeletal relationship, 20.0% (n = 6) with Class II, and 13.3% (n = 4) with Class III (Table 1). Each patient underwent four standardized sEMG assessments across the course of early orthodontic treatment (T0–T3), yielding a total of 1440 recordings.

During the broad smile expression, no statistically significant changes were observed in the mean peak or minimum sEMG amplitudes across any of the examined muscle groups between the four time points (all *p* > 0.05). Muscle activation remained stable throughout treatment during this movement, suggesting that broad smiling is not influenced by the leveling and alignment phase of orthodontic therapy.

**Figure 3:**
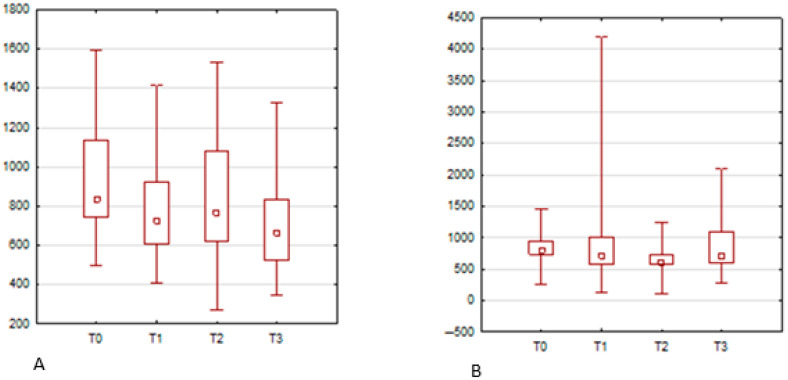
Illustrates box-and-whisker plots showing changes in mean peak sEMG activity of the orbicularis oris during forward lip protrusion. On the left side (**A**), a significant increase was observed at T3 compared to T0. On the right side (**B**), significant increases were noted at T1 and T2 versus T0.

**Figure 4:**
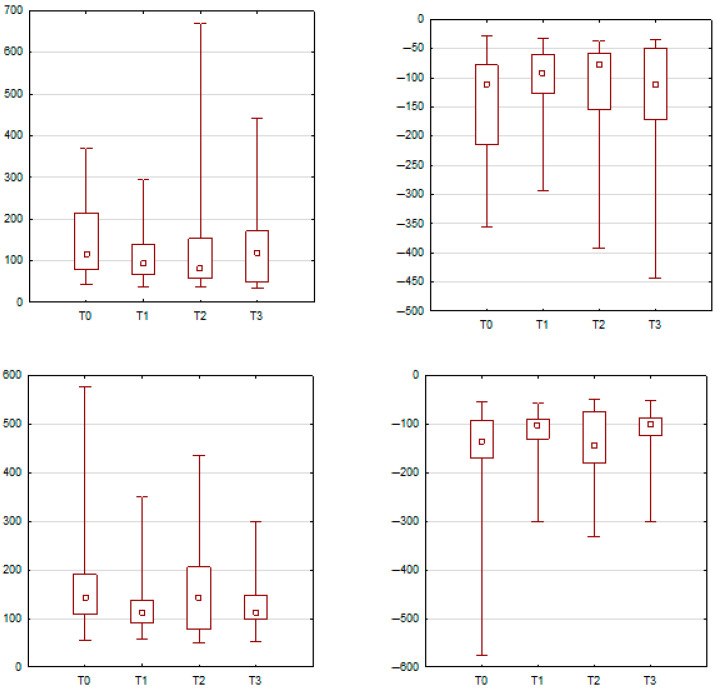
sEMG changes in two additional muscle groups during forward lip protrusion. Part A presents zygomaticus major activity (**right side**), which increased significantly at T1, T2, and T3 compared to T0. Part B shows orbicularis oculi (**right side**) activity, which was significantly higher at T1 and T3 relative to baseline.

In contrast, forward lip protrusion elicited several significant changes in muscle activity. For the orbicularis oris muscle on the left side, mean peak amplitude was significantly higher at T3 compared to T0 (*p* = 0.0012), while the minimum amplitude also increased significantly at T2 and T3 compared to baseline. On the right side, the orbicularis oris demonstrated significantly elevated mean peak values at T1 (*p* = 0.0039) and T2 (*p* < 0.001) relative to T0. The zygomaticus major muscle on the right showed significantly increased peak sEMG activity at T1, T2, and T3 compared to baseline values (*p* = 0.0144, 0.0109, and 0.0061, respectively). Similarly, the orbicularis oculi on the right side showed significantly higher peak amplitude at T1 and T3 relative to T0 (*p* = 0.0121 and *p* = 0.0043). These findings are presented in Figure 3 and Figure 4 and detailed in Table 2 and Table 3.

**Figure 5:**
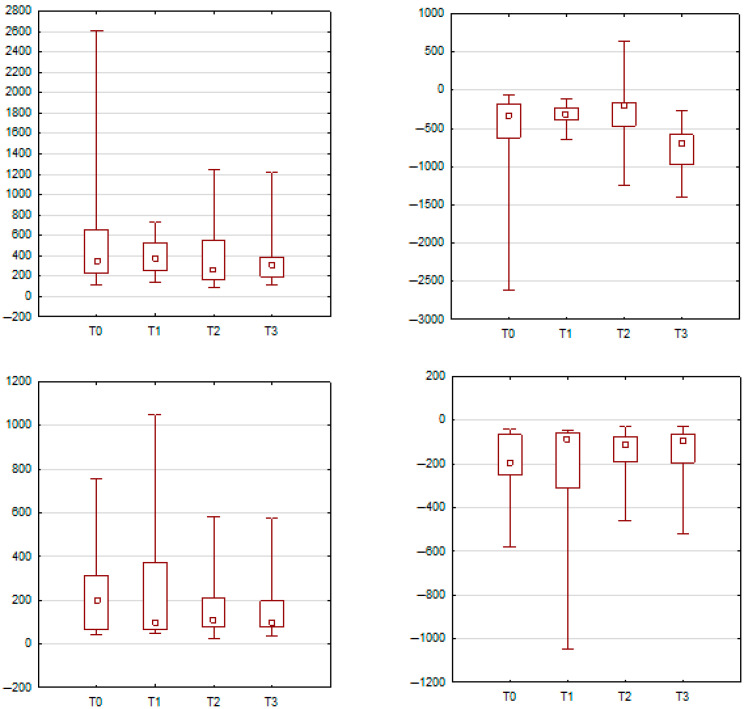
Changes during nasal strain. In part A, the orbicularis oris (**right side**) demonstrated elevated peak and minimum amplitudes at T2 and T3. Part B shows a significant increase in zygomaticus major (**right side**) activity at T1 and T2 compared to T0.

**Figure 6:**
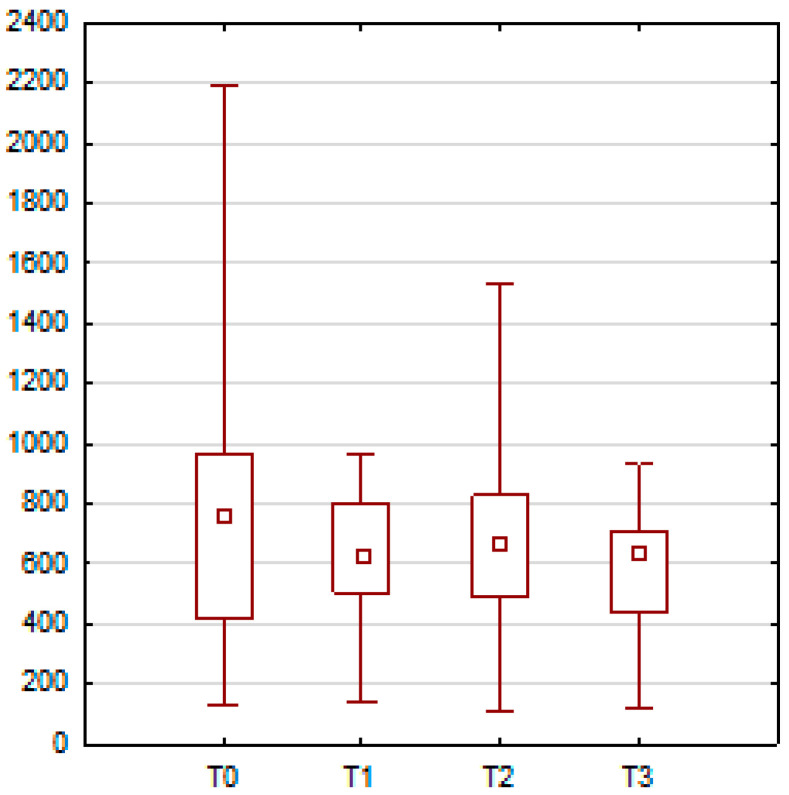
The mean peak activity of the levator labii superioris alaeque nasi (**right side**) during nasal strain. A significant increase in activity was observed at T1, T2, and T3 compared to T0.

During nasal strain, significant neuromuscular changes were also observed. The orbicularis oris muscle on the right side exhibited significantly elevated peak and minimum amplitudes at T2 and T3 when compared to T0 (*p* = 0.0011 and *p* = 0.0053, respectively). The right-sided zygomaticus major also demonstrated significantly higher peak activity at T1 and T2 compared to baseline (*p* = 0.0063 and *p* = 0.0365). Moreover, the levator labii superioris alaeque nasi muscle on the right side showed significantly increased peak amplitude at all post-treatment time points (T1, T2, and T3) relative to T0, with *p*-values < 0.001 for each comparison. These results are illustrated in Figure 5 and Figure 6 and summarized in Table 4 and Table 5.

**Figure 7:**
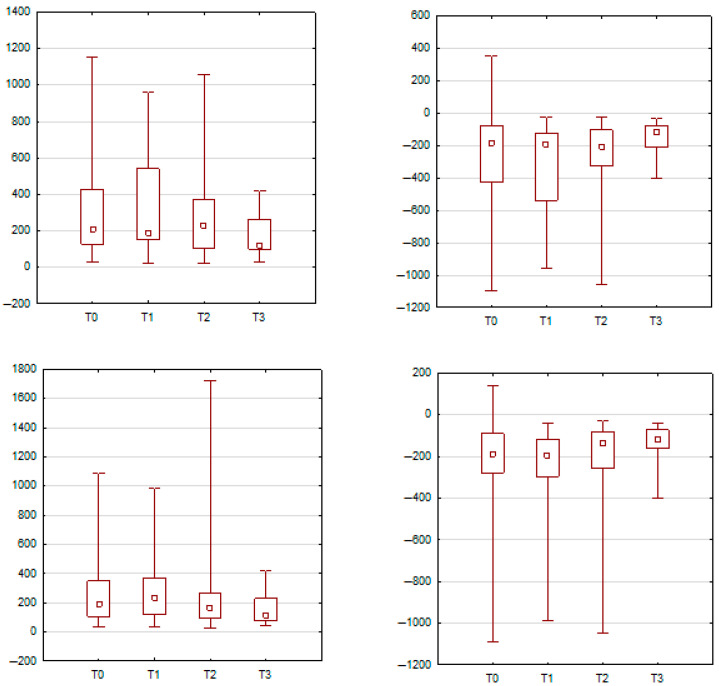
Changes in orbicularis oris activation during eye closure. Both the left and right sides showed significantly higher peak and minimum amplitudes at T3 compared to earlier time points.

During eye closure, the orbicularis oris muscle on both the left and right sides demonstrated a statistically significant increase in both peak and minimum sEMG amplitudes at T3 in comparison to T0, T1, and T2 (*p* < 0.01). However, no significant changes were observed in the activity of the orbicularis oculi or zygomaticus major muscles during this expression. These findings are presented in Figure 7 and Table 6.

In summary, the leveling and alignment phase of orthodontic treatment was associated with significant increases in sEMG activity during specific facial expressions, particularly forward lip protrusion, nasal strain, and eye closure. The most pronounced changes were observed in the orbicularis oris, zygomaticus major, and levator labii superioris alaeque nasi muscles, predominantly on the right side. These results support the hypothesis that mimic muscles undergo dynamic neuromuscular adaptation during early orthodontic treatment.

## 4. Discussion

This study investigated dynamic changes in facial expression muscle tone during the leveling and alignment phase of fixed appliance orthodontic treatment using surface electromyography (sEMG). The concept was inspired by the pilot study conducted by Rodríguez et al. [12], who assessed changes in masseter muscle activity during different phases of orthodontic therapy. Their study underscored two main limitations: the heterogeneity of individual treatment plans, which limits standardization, and the small sample size, which constrained the generalizability of their findings. Despite these challenges, they demonstrated that the masseter muscle undergoes measurable changes in tone during treatment.

Our findings extend these observations by focusing on a broader range of facial expression muscles, which have not been comprehensively studied in the context of orthodontic therapy. Similar changes in muscle tone, particularly in response to orthodontic force application, have been documented in studies by Park et al. [13], Farronato et al. [14], Frongia et al. [15], Ko et al. [16], and Kubota et al. [17], especially among patients undergoing combined orthodontic and surgical treatment. However, these studies concentrated primarily on the masticatory system. To the best of our knowledge, this is the first study to systematically analyze sEMG activity in multiple groups of mimic muscles throughout a defined stage of orthodontic treatment, which makes our work novel in the orthodontic literature.

The utility of sEMG in dentistry has been widely acknowledged, as outlined in the reviews by Dyduch et al. [18] and Śpiewok et al. [19]. These authors emphasize its value in detecting muscular hyperactivity and hypoactivity, evaluating resting mandibular posture, assessing right-left muscular asymmetries, and guiding parafunction management. The advantages of sEMG include non-invasiveness, patient comfort, and the ability to simultaneously monitor multiple muscle groups. Nonetheless, its use in facial muscle assessment is limited by certain technical challenges. Most notably, sEMG is susceptible to motion artifacts, which may compromise signal reliability during facial movement. Another limitation relates to the absence of amplitude normalization. Although we intentionally avoided MVC-based scaling to preserve signal integrity and avoid artifacts from inconsistent facial contractions, this decision limits the comparability of absolute amplitude values both within and between subjects. Consequently, our results are best interpreted as relative changes across time within the same muscle rather than direct comparisons between muscles or individuals. Future studies may consider alternative normalization strategies such as submaximal standard tasks or percentile-based scaling. Additionally, in patients with dysfunctional swallowing patterns, such as persistent infantile swallowing, co-activation of adjacent muscles (e.g., orbicularis oris during orbital movement) may lead to signal contamination.

Anatomical considerations further complicate the interpretation of sEMG data in the facial region. Facial expression muscles exhibit intricate fiber interdigitation and share common dermal attachments, forming a functionally integrated network. As a result, the pure isolation of individual muscles via surface electrodes is inherently difficult. In our study, co-activation was observed across muscle groups regardless of the specific facial expression performed, which supports this interconnected anatomical behavior [20]. We therefore acknowledge that while sEMG offers valuable insights into general trends in muscle activity, needle electromyography (EMG) would likely provide greater specificity in evaluating the behavior of individual mimic muscles. Although this study did not include direct clinical outcome measures such as standardized photographic analysis, facial esthetic evaluations, or patient-reported assessments, the observed neuromuscular changes may have implications for perioral function and soft tissue dynamics. Increased activity in muscles such as the orbicularis oris and zygomaticus major could influence lip posture, smile esthetics, or facial symmetry during treatment. Future research should aim to correlate sEMG findings with objective clinical outcomes, including three-dimensional facial imaging, smile analysis, or esthetic satisfaction surveys. Such integrative approaches would help determine whether the detected electromyographic adaptations translate into visible or functionally relevant changes from the patient’s perspective. A key methodological limitation is the absence of a control group. The inclusion of a non-treatment cohort could strengthen causal inference and help distinguish orthodontic-induced neuromuscular changes from natural variability. However, ethical and logistical barriers precluded this implementation in the present study. Future studies should consider a matched control group to enhance the internal validity of observed effects.

Age-related physiological processes may also influence muscle tone. Sarcopenia, the gradual loss of muscle mass with age, can lead to diminished facial mobility, increased baseline tension, and reduced expressiveness. These factors may contribute to the formation of static wrinkles from previously dynamic ones [20]. Although our cohort was relatively young (aged 20–31 years), we investigated whether subtle age-related differences were evident within this range. No significant associations were found between age and muscle tone during broad smile or lip protrusion tasks. However, our observations are consistent with the findings of George et al. [21], who noted that patients closer to 30 years of age exhibited greater activation of the orbicularis oris during nasal strain and increased levator labii activity during eye closure. These trends may be attributable to early biomechanical adaptations in muscle length or recruitment patterns, as previously reported.

The primary limitation of our study is the relatively small and demographically homogeneous sample, which included only female participants. This selection was intentional to reduce biological variability; however, it limits the generalizability of the findings to the broader population. Sex-based differences in facial muscle anatomy, tone, and activation patterns may significantly influence sEMG results. Previous studies have indicated that male subjects tend to exhibit greater baseline muscle thickness and mass, as well as different recruitment strategies during facial expression tasks [22]. Furthermore, hormonal influences and sexual dimorphism in soft tissue distribution can contribute to variation in mimic muscle response. Therefore, the restriction to female participants in this study, while methodologically justified to enhance internal consistency, precludes extrapolation of our findings to male patients. Future research should include male participants to evaluate whether similar neuromuscular adaptation patterns occur across sexes. Future studies should include a larger, sex-balanced cohort and consider long-term follow-up beyond the alignment phase. In addition, combining sEMG with three-dimensional facial imaging and functional assessments may further enhance our understanding of how orthodontic forces interact with soft tissue dynamics. Furthermore, the six-month observation window captures only the initial phase of orthodontic therapy. While this is a period of rapid neuromuscular adaptation, it may not fully reflect the long-term behavior of mimic muscles. Extended follow-up across the full duration of treatment and post-treatment retention is necessary to validate whether these early changes persist or normalize over time.

## 5. Conclusions

This study demonstrated that the leveling and alignment phase of fixed appliance orthodontic treatment is associated with significant neuromuscular adaptations in facial expression muscles. The most prominent changes were observed during forward lip protrusion and nasal strain, particularly in the orbicularis oris, zygomaticus major, and levator labii superioris alaeque nasi muscles. These findings highlight the importance of monitoring soft tissue function during early orthodontic therapy, as muscular responses may influence facial symmetry and esthetics.

Future studies involving larger, more diverse cohorts and longer follow-up are warranted to determine whether these electromyographic changes have lasting clinical relevance.

## Figures and Tables

**Table 1:** Baseline characteristics of the study cohort (n = 30). Continuous data are presented as mean ± standard deviation (SD), range, and median with inter-quartile range (Q1–Q3). Categorical variables are reported as number of participants and corresponding percentages.

Variable	n	Mean ± SD	Range	Median (Q1–Q3)	Units/Notes
Age	30	24.6 ± 3.0	20–31	24.0 (23.0–27.0)	years
Skeletal Class I	20	–	–	–	n (%) = 66.7%
Skeletal Class II	6	–	–	–	n (%) = 20.0%
Skeletal Class III	4	–	–	–	n (%) = 13.3%

**Table 2:** Bonferroni-adjusted *p*-values (left/right) for pair-wise comparisons of the mean peak RMS sEMG amplitude (% of baseline) of the orbicularis oris during lip protrusion across four treatment stages (T0–T3). Significant differences (*p* < 0.05) are highlighted in bold.

	T0	T1	T2	T3
T0	–/–	0.088/0.004	0.161/<0.001	0.001/0.157
T1	0.088/0.004	–/–	0.754/0.157	0.108/0.128
T2	0.161/<0.001	0.754/0.157	–/–	0.056/0.004
T3	0.001/0.157	0.108/0.128	0.056/0.004	–/–

Values are shown as “left side/right side”. RMS = root mean square; % = percentage of baseline activity.

**Table 3:** Bonferroni-adjusted *p*-values for pair-wise comparisons of mean peak RMS sEMG amplitude (% of baseline) during lip protrusion across four treatment stages (T0–T3). Values <0.05 (statistically significant) are shown in bold.

	T0	T1	T2	T3
Zygomaticus major (right)				
T0	–	0.014	0.011	0.006
T1	0.014	–	0.917	0.756
T2	0.011	0.917	–	0.836
T3	0.006	0.756	0.836	–
Orbicularis oculi (right)				
T0	–	0.012	0.323	0.004
T1	0.012	–	0.120	0.715
T2	0.323	0.120	–	0.056
T3	0.004	0.715	0.056	–

Values are presented as right-side data only. RMS = root mean square.

**Table 4:** Bonferroni-adjusted *p*-values (rounded to three decimals) for pair-wise comparisons of mean peak RMS sEMG amplitude (% of baseline) during nose-strain expression at four treatment stages (T0–T3). Significant differences (*p* < 0.050) are shown in bold.

	T0	T1	T2	T3
Orbicularis oris (right)				
T0	–	0.172	0.001	0.005
T1	0.172	–	0.047	0.142
T2	0.001	0.047	–	0.598
T3	0.005	0.142	0.598	–
Zygomaticus major (right)				
T0	–	0.502	0.006	0.091
T1	0.502	–	0.036	0.303
T2	0.006	0.036	–	0.280
T3	0.091	0.303	0.280	–

RMS = root mean square.

**Table 5:** Bonferroni-adjusted *p*-values (rounded to three decimals) for pair-wise comparisons of mean peak RMS sEMG amplitude (% of baseline) of the levator labii superioris alaeque nasi—right side—during nose-strain expression (T0–T3). Significant differences (*p* < 0.050) are shown in bold.

	T0	T1	T2	T3
T0	–	0.090	0.393	<0.001
T1	0.090	–	0.393	0.035
T2	0.393	0.393	–	0.004
T3	<0.001	0.035	0.004	–

RMS = root mean square.

**Table 6:** Bonferroni-adjusted *p*-values (three-decimal rounding) for pair-wise comparisons of mean peak RMS sEMG amplitude (% of baseline) during eye closure. Values are shown as “left side/right side”: orbicularis oculi (left) and orbicularis oris (right). Significant differences (*p* < 0.050) are highlighted in bold.

	T0	T1	T2	T3
T0	–/–	0.746/1.000	0.162/0.351	<0.001/0.006
T1	0.746/1.000	–/–	0.281/0.351	<0.001/0.006
T2	0.162/0.351	0.281/0.351	–/–	0.015/0.064
T3	<0.001/0.006	<0.001/0.006	0.015/0.064	–/–

RMS = root mean square.

## Data Availability

The data presented in this study are available on request from the corresponding author. The data are not publicly available due to privacy restrictions.
